# Ecosystem services and connectivity in spatial conservation prioritization

**DOI:** 10.1007/s10980-016-0446-y

**Published:** 2016-09-26

**Authors:** Aija S. Kukkala, Atte Moilanen

**Affiliations:** 1 Department of Biosciences, University of Helsinki, PO Box 65, 00014 Helsinki, Finland; 2 Department of Geosciences and Geography, University of Helsinki, PO Box 68, 00014 Helsinki, Finland

**Keywords:** Accessibility, Biodiversity, Complementarity, Green infrastructure, Optimization, Spatial interactions, Systematic conservation planning, Trade-offs, Zonation software

## Abstract

**Context:**

Spatial conservation prioritization (SCP) concerns, for example, identification of spatial priorities for biodiversity conservation or for impact avoidance in economic development. Software useable for SCP include Marxan, C-Plan and Zonation. SCP is often based on data about the distributions of biodiversity features (e.g., species, habitats), costs, threats, and/or ecosystem services (ES).

**Objectives and methods:**

At simplest ES can be entered into a SCP analysis as independent supply maps, but this is not very satisfactory because connectivity requirements and consequent ideal spatial priority patterns may vary between ES. Therefore, we examine different ES and their connectivity requirements at the conceptual level.

**Results:**

We find that the ideal spatial priority pattern for ES may differ in terms of: local supply area size and regional network requirements for the maintenance of ES provision, for flow between provision and demand, and with respect to the degree of dispersion that is needed for ES provision and access across different administrative regions. We then identify existing technical options in the Zonation software for dealing with such connectivity requirements of ES in SCP.

**Conclusions:**

This work helps users of SCP to improve how ES are accounted for in analysis together with biodiversity and other considerations.

## Introduction

Spatial conservation prioritization (SCP) concerns identification of spatial priorities for expansion of conservation area networks, identification of areas for impact avoidance in economic development, allocation of habitat restoration and biodiversity offsetting, and other forms of spatial conservation resource allocation. It is a quantitative analytical step that is often utilized within a broader operational framework for the implementation of conservation, such as systematic conservation planning (Margules and Pressey 2000). SCP analyses are often carried out by special software, originally designed for solving reserve selection problems, such as Marxan (Ball and Possingham 2000; Ball et al. 2009), C-Plan (Pressey 1999) and Zonation (Moilanen et al. 2005; Di Minin et al. 2014). SCP is most commonly based on data about the distributions of species and habitat types, but additional information about costs, threats, connectivity or ecosystem services (ES) is sometimes used (Kullberg and Moilanen 2014). At simplest, ES can be entered into SCP analysis as independent supply maps, but this is not very satisfactory because ideal spatial patterns may be different for different ES, or even for the same ES in different locations.

Many SCP approaches and methods were first developed for biodiversity conservation. Already almost 10 years ago, Chan et al. (2006) and Egoh et al. (2007) put forward the conceptual argument that prioritization for ES could be integrated and implemented using SCP software packages such as Marxan and C-Plan. While some studies have investigated spatial prioritization of ES, most of these have focused on one or a few ES only (Chan et al. 2006, 2011; Izquierdo and Clark 2012; Casalegno et al. 2014; Schröter et al. 2014; Nin et al. 2016). Several have also investigated (spatial) coincidence between biodiversity and ES (Costanza et al. 2007; Mace et al. 2012; Reyers et al. 2012; Cimon-Morin et al. 2013). Some ES have been described as “conservation compatible” meaning that the presence of the service could be regarded as an additional argument for conservation (Chan et al. 2011; Schröter and Remme 2016). While a few studies have considered the methodological aspects of spatial prioritization of ES (Cimon-Morin et al. 2014; Schröter and Remme 2016; Snäll et al. 2016), none of these studies have been specific on different ways of treating connectivity in spatial prioritization for ES.

Spatial prioritization without any explicit connectivity effects can provide useful summary information about distribution patterns of ecosystem services. Nevertheless, connectivity, spatial interactions between landscape elements, should be accounted for as a primary consideration in spatial ecology and prioritization. While connectivity has been proposed as important for spatial prioritization of ES (Cimon-Morin et al. 2013; Snäll et al. 2016), there are only a few operational examples of how to actually implement such analyses. Chan et al. (2011) found that their ES priority distribution by Marxan consisted of several small patches that are unlikely to be realistically implementable as conservation areas. Like species populations need area for persistence, ES may have minimum local area requirements for provision. For example, recreational value is only produced by a large enough area. ES such as pollination may require linkage (flow, accessibility) between provision and demand (Fisher et al. 2009; Bagstad et al. 2013; Burkhard et al. 2014), which complicates spatial analysis (Luck et al. 2012; Cimon-Morin et al. 2014; Serna-Chavez et al. 2014). Schröter and Remme (2016) found that hotspot methods used for identifying ES priority sites can lead to spatial scattering, implying that some hotspot methods may not be suitable for identifying priority sites for ES conservation. Furthermore, there is the consideration that not all ES may be able to coexist: in particular provisioning services (such as timber harvesting or hunting) may be at odds with services such as recreation or biodiversity conservation as a cultural service (Price et al. 2016). Overall, it seems that treatment of ES in SCP could be more even complicated than treatment of multiple species distributions.

Data resources based on remote sensing, land surveys, and spatial modeling have become increasingly available, and there have been several quantitative and qualitative mapping efforts for regional ES provision (Burkhard et al. 2012; Maes et al. 2012a). Hence, spatial data are becoming more common, which facilitates increasingly realistic integration of ES in SCP. Software tools available for spatial prioritization (Moilanen et al. 2009; Di Minin et al. 2014) also enable relatively routine application of SCP conditional on the availability of adequate data. SCP concepts, such as complementarity and connectivity, are migrating to ES studies (Cimon-Morin et al. 2016), demonstrating a clear possibility and need for improved linkage of SCP and ES. Therefore, we here describe a new typology of ES and connectivity appropriate for use in SCP, and identify operational alternatives for including ES in the Zonation spatial prioritization framework (Moilanen et al. 2005, 2014), which has multiple pre-existing features available for the treatment of connectivity (summarized by Lehtomäki and Moilanen 2013). The Zonation software produces a hierarchical prioritization of the landscape based on data about the occurrence levels of biodiversity features and possibly ES, costs and threats in sites. When developing the priority ranking, concepts and principles such as connectivity, complementarity, and balance between features are accounted for.

### Typology of connectivity of ES and technical solutions for inclusion in spatial prioritization

Connectivity is the manner or extent to which species or resources disperse and interact across landscapes (Biggs et al. 2012; Mitchell et al. 2013; Ng et al. 2013). It is important for ecological functions underlying many ecosystems services (de Groot et al. 2002; Haines-Young and Potschin 2009; Mitchell et al. 2013, 2015). ES maintenance, especially for regulating or supporting services, relies on the mobility of organisms and ecological flows in the landscape (Mitchell et al. 2013). Highlighting growing attention on connectivity of ES, it has been recognized as one of the key principles in the EU’s green infrastructure strategy: areas that provide ES should also be interconnected (European Commission 2013). However, while aggregated priority pattern could be ideal for some ES, in some cases dispersion can improve ES provision and accessibility (Casalegno et al. 2014; Mitchell et al. 2015).

**Fig. 1:**
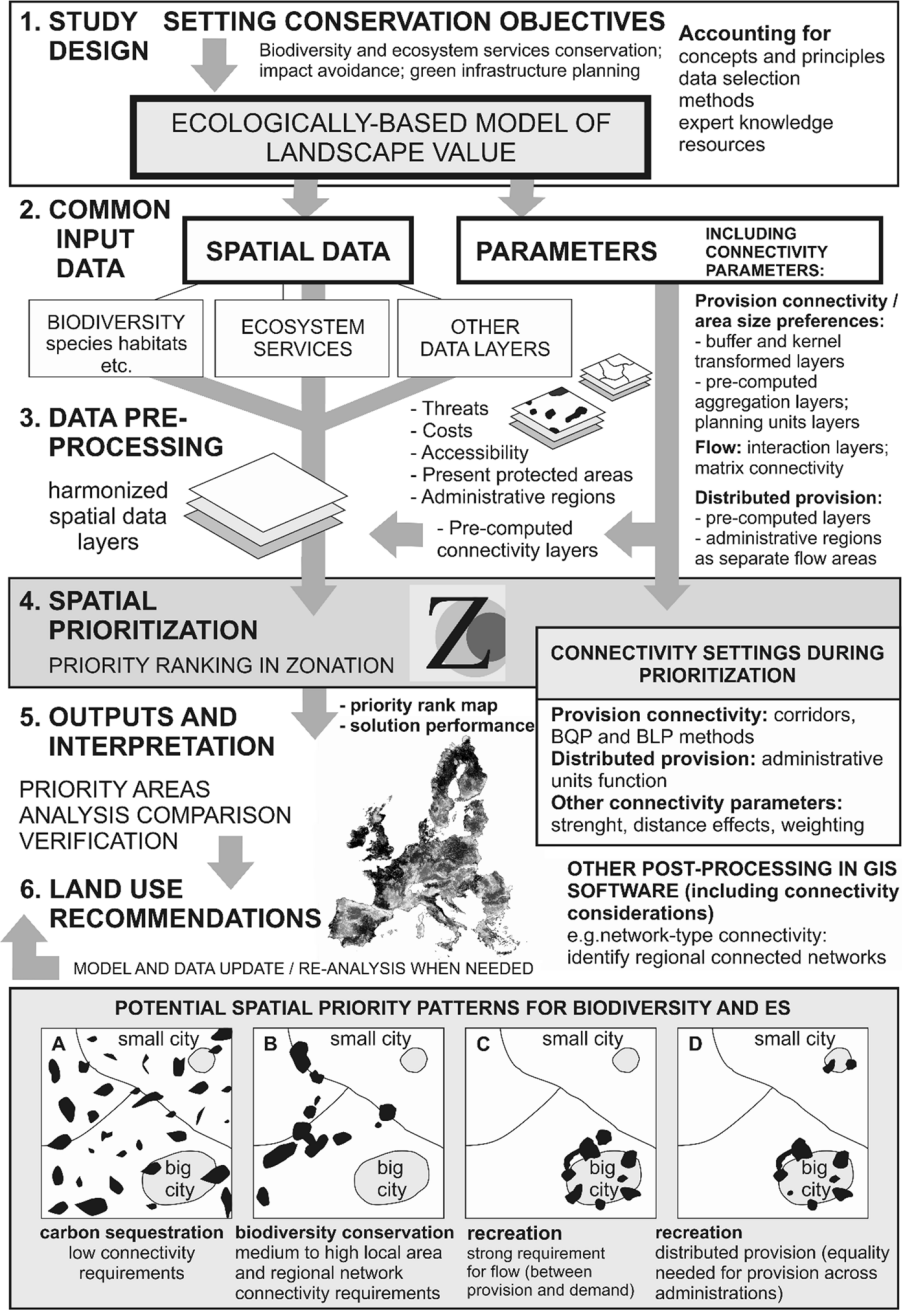
Schematic illustration of the process of spatial prioritization, with entry points for connectivity considerations marked, linking to Table 1. Main options for dealing with connectivity considerations include in data preparation and preprocessing (either externally or by Zonation), or during the computational prioritization run itself. The strength and spatial scale of a connectivity response can typically be specific by parameters (see Lehtomäki and Moilanen 2013). Limited options for accounting for connectivity exist at the step where priority rank maps are interpreted and post-processed for decision making. Spatial prioritization methods can simultaneously balance the needs of many biodiversity features or ES, aiming at solutions that combine different spatial needs, illustrated by panels **a**–**d**

Table 1 describes our typology of connectivity for ES and summarizes how such connectivity requirements may be incorporated into SCP using Zonation and technical solutions available in it. Our main classification is between (i) connectivity needed for ES provision, (ii) connectivity needed for ES flow between supply and demand, and (iii) dispersed supply and equitable accessibility across administrations, which are discussed in separate sections below. These same techniques or variants of them can be used in other SCP software depending on the types of connectivity responses that have been made available. Figure 1 summarizes how ES enter SCP in the Zonation software along with connectivity requirements; it also schematically introduces examples of potential priority patterns for different ES. Table 2, below, discusses different connectivity requirements from the perspective of different classes of ES.

**Table 1:** Technical solutions for inclusion of ES and their connectivity requirements into spatial conservation prioritization (SCP)

Connectivity characteristics and examples	Possible solutions for integration in SCP
Low connectivity requirements	
The ES can be provided locally in small areas. Can be transported over distances when needed. Example: Carbon, or other ES with weak/uncertain spatial effects	Simply enter grid into SCP as a feature. Allow it to become protected with spatial pattern most governed by requirements of other features. (pre-processing)
Provision connectivity: aggregation and local minimum area requirements	
Service is not provided by too small areas, because of e.g. edge effects, minimum population sizes, space needs of dynamic processes. Also logistical/administrative requirements may imply minimum area size. This is a common requirement; examples include recreation, ground water, and biodiversity conservation	Many solutions for inducing aggregated priorities: (i) Connectivity techniques such as distribution smoothing (pre-processing), corridor connectivity (prioritization), boundary quality penalty (prioritization), or boundary length penalty (prioritization), with a suitably chosen spatial scales (see Lehtomäki and Moilanen 2013) (ii) Can also be implemented by using suitably large predefined spatial planning units in analysis (pre-processing)
Provision connectivity: regional connected networks desirable	
Maintenance of large-scale spatial dynamical processes. Example: area networks for the maintenance of biodiversity or pollinators	Comparatively difficult to implement due to technical complications and lack of data about connectivity effects: no standard SCP methodology exists for simultaneous design of networks for many partially conflicting factors. Local area requirements (above) contribute to regional connectivity. (i) Pre-computed connectivity layers can be entered into analysis to give priority to areas assumed important for network connectivity (pre-processing) (ii) Alternatively, regional connected networks can be detected from Zonation output maps in post-processing
ES flow: proximity between demand and supply needed	
This consideration is separate from, and can be combined with other connectivity components. Proximity between demand and supply can be regarded at local, regional, or global scales. Common requirement with ES. For example, recreation, or pollination	(i) Connectivity interaction (pre-processing) at given spatial scale (Rayfield et al. 2009) (ii) When spatial overlap is required, add product of demand and supply layer into analysis (pre-processing) (iii) Via multi-feature connectivity (matrix connectivity; Lehtomäki et al. 2009) to promote areas with compatible land use mixes (iv) Enter separate feature layer for each ES flow area (pre-processing)
Distributed ES provision	
Many countries, regions or other administrations wish to maintain their own ES, implying need for large-scale distributed priorities. Applies to most ES, such as recreation, ground water, cultural services. This consideration is separate from, and can be combined with other connectivity components.	(i) Use administrative units (ADMU) (Moilanen and Arponen 2011) (prioritization) (ii) Enter different feature layers for different areas (pre-processing) (iii) Combination of the above (iv) Use special dispersal kernel with lowest connectivity at middle distances (pre-processing)

For each solution, we indicate where in the workflow of SCP the solution is implemented: data pre-processing, prioritization, or post-processing (Fig. 1)

**Table 2:** Illustrative examples of connectivity considerations for selected ES, following the most well-known classification into provisioning, regulating, and cultural ecosystem services (CICES 2016)

ES category	Local area requirements	Regional network-type connectivity	Demand for ES flow	Need for distributed access
Provisioning services	Maintenance of ecosystem processes may imply minimum area size for successful ES provision; e.g. hunting, fishing Also logistical considerations may favor larger areas: e.g., cultivated crops e.g. ground water, whole ground water area requires maintenance	Maintenance of viable (ecological) networks needed for provisioning services that depend on biodiversity or ecosystem processes and function e.g. anything depending on biodiversity; river systems	Logistical requirements between ES provision and beneficiaries: low to high requirements e.g. cultivated crops (accessibility is important, although commonly transported long distances) e.g. wild food, often utilized in situ, flow only at short distances	Considerations of security or equitable provision imply distributed supply e.g. drinking water
Regulation and maintenance services	Large variation in local area requirements between different ES e.g. carbon sequestration, low local area requirements e.g. pollination, can be provided by smallish but high quality areas e.g. flood regulation, large enough areas required	e.g. biodiversity-dependent services including pollination: maintenance of (meta)populations needed via sufficiently dense networks of populations e.g. flood regulation, maintenance of landscape quality at catchment scale	Large variation. e.g. carbon sequestration, low local flow requirements e.g. air quality regulation, high local & regional-scale requirements e.g. pollination, high localized flow requirement	Largely same as above. e.g. air quality regulation: service desirable for all people e.g. flood regulation, service desirable for all people in flood-prone environments
Cultural services	Requirement highly variable e.g. sense of place, no specific area requirement e.g. green areas for recreation need to be large enough	Variable requirement e.g. sense of place: networks not needed necessarily e.g. outdoor recreation: connected network of green areas may be preferable	Requirement for flow is high: cultural services needed where there are people e.g. recreation; accessibility of local recreational areas	High requirement for distributed supply and access. Globally aggregated supply very unsatisfactory

Note that multiple connectivity requirements (provision, flow & distributed access) can apply to an individual ES simultaneously

#### Provision connectivity: aggregation requirements for maintenance of ES provision

The first common requirement in spatial prioritization is to account for aggregation and local area size requirements. ES require sufficiently large areas for underlying ecological processes to operate (Kremen 2005), and some services, such as outdoors recreation, typically cannot be provided by very small areas (Table 2). There are many solutions in SCP software to promote connectedness and locally aggregated areas, including the boundary length penalty, which has also been applied in some ES studies that used the Marxan approach (e.g. Chan et al. 2006; Izquierdo and Clark 2012). Several other connectivity techniques that have feature-specific connectivity scales, such as distribution smoothing or the boundary quality penalty are applicable as well (Table 1).

Whilst area size is a fundamental building block of connectivity, it is a component of more general regional network connectivity, which may be needed for the maintenance of the species and ecological processes that support provision of ES (Table 2). For example, biodiversity conservation is well known to benefit from regionally connected conservation area networks (Hanski 1998; Rayfield et al. 2011). Some ES, such as pollination, may have both local area size and regional network connectivity requirements. Of the types of connectivity discussed here, regional connectivity is operationally perhaps the most difficult one to account for, because its significance varies between individual ES and the ecosystems and species that underlie them. Izquierdo and Clark (2012) accounted for landscape elements that had been a priori designated as important for regional connectivity by favoring selected planning units for inclusion in corridors via the Marxan penalty factor.

#### ES flow between provision and demand

ES beneficiaries are often located elsewhere than ES provision sites, leading to the second major type of connectivity relevant for ES (Table 1). It is a common and important requirement that there is proximity (effectively connectivity) between ES provision and demand, which is often called ES flow (Bagstad et al. 2013; Serna-Chavez et al. 2014). Depending on ES, flow areas can be regarded as local, regional or global (Cimon-Morin et al. 2014; Table 2). Many ES, such as outdoors recreation or pollination, benefit from clear proximity between supply and demand. Indeed, flow has been seen as a fundamental characteristic of ES (Costanza 2008; Bennett et al. 2009; Fisher et al. 2009), especially for cultural services (Table 2). Cimon-Morin et al. (2013) have previously suggested that in regions dominated by humans, ES priority areas may be identified based on biophysical potential alone (because demand is always nearby). In less populated landscapes, on the other hand, where demand for ES is low, ES quantification could primarily be based on the distribution of beneficiaries.

One way of implementing flow in prioritization is to use the connectivity interaction feature in Zonation, which emphasizes areas where two features (here ES provision and demand) occur nearby (or overlapping) each other as specified via a spatial scale parameter (Rayfield et al. 2009). Another option is to treat each ES provision-demand flow area as a separate feature layer that requires representation, thereby replacing conceptual elegance with a straightforward but versatile brute-force computational strategy (Verhagen et al. 2016, pers. comm.). Note that sometimes one might need to account for the direction of connectivity, because ES production-consumption flows can have different directions. For example, pollination has an omnidirectional flow zone whereas water flow regulation is influenced by directional flow (Cimon-Morin et al. 2013).

#### Distributed ES provision

The ES priority pattern can differ also with respect to the degree of dispersion (as opposed to aggregation) that is needed to guarantee ES provision and accessibility equitably across different administrations (Table 1). For example, green area in a city provides recreation services for residents by a locality-dependent non-transferable service (Fig. 1). In other words, most countries, cities, or regions wish to maintain some of their own ES. There are options for dealing with dispersion requirements of ES. One can simply enter separate feature layers for different areas or use the so-called administrative units analysis in Zonation (Moilanen and Arponen 2011), which utilizes an arbitrary user-chosen division of the landscape to enforce distributed provision of the spatial features of interest (Table 1).

#### Other considerations

There may be additional considerations not treated in Table 1. If flow between provision and demand is to be accounted for, there is the question of whether to prioritize based on known present demand or whether one should also prepare for unexpected future changes. If the future perspective is taken, sites that are the most important for ensuring the continuous supply of services should receive elevated priority irrespective of present demand (Cimon-Morin et al. 2013, 2014). This kind of place-based approach sets demands also for the data used in setting priorities, and increases the need for combining multiple data sources.

When using multiple biodiversity features and ES, and their connectivity requirements in the same analysis, it becomes necessary to specify the relative weightings of features. In the case of ES, the weight given to an ES supply or demand distribution or to a connectivity consideration should depend, e.g., on the value given to the service, the quality of data, and the reliability of assumptions about connectivity responses. See Lehtomäki and Moilanen (2013) and Lehtomäki et al. (2016) for discussion about weight-setting in Zonation. Note that the data resolution also has direct implications for spatial prioritization. For example, if spatial resolution is 10 × 10 km grid cells, local area and connectivity requirements may be automatically met inside individual grid cells, while analysis using a 20 × 20 m resolution would definitely require setting connectivity parameters, because the small grid cells are inevitably dynamically linked with their neighbors.

### Interactions between ES

The connectivity effects discussed above are relevant when ES are considered on their own, independently from each other, and irrespective of other features such as biodiversity. Additional complications are introduced when it is acknowledged that there may also be synergies or tradeoffs to take into account (Power 2010; Haase et al. 2012; Maes et al. 2012b). While multi-functionality of ES in prioritization is automatic in complementarity-based Zonation, conflicts between ES are likely to occur. It is hard to have many things connected at various scales and dispersed at the same time. Increasing the supply of one ES can either enhance or hamper the supply of others (Bennett et al. 2009; Maskell et al. 2013). Further complicating issues, these interactions can be considered both locally for ES occurring in an overlapping manner and between ES that occupy neighboring areas.

It may be a challenge to integrate synergies and trade-offs into SCP. Effectively, these are nonlinear interactions, which are not automatically accounted for in SCP, which is most often based on analysis of static patterns. While mathematical solutions to interaction matrices might be available via e.g. analogue to species in community ecology (McGill et al. 2006), there is the additional consideration that SCP methods need to be operational when there are many thousands of features in analysis and the count of spatial elements goes up to tens of millions of grid cells (e.g., Pouzols et al. 2014). What then can be said about synergies or trade-offs between ES in spatial prioritization?

First, there is the trivial case when there is no interaction between the ES in question. This would be the case for example between aesthetic value and ground water. In this case, one can enter layers as independent features into analysis.

Second, there is the case of synergy or positive feedback. This could be the case for example between vegetation and ground water: above ground conservation may help maintain water quality. In Zonation, a positive spatial interaction can be modeled via the interaction connectivity technique (Rayfield et al. 2009). Another method for implementing such an effect is via data pre-processing: a new layer can be derived (and entered into analysis) as a product (interaction) of the two ES layers in question. Effectively, synergies between features are not a major problem for analysis, as the locations where both features occur will tend to become emphasized as a natural outcome of complementarity-based analysis and this effect can be further strengthened via the addition of the positive interaction layer. For example, many regulating and cultural ES have none or synergistic relationships with each other (Bennett et al. 2009).

Third, there is the case of negative interactions between ES. This could be the case for example between timber harvesting and carbon sequestration. Negative interactions could be relevant also when anticipating future conflicts between a green infrastructure network and competing land uses. As above, the interaction connectivity technique can be used to model a negative interaction (Rayfield et al. 2009), reducing occurrence levels of ES where they occur near each other. Similarly, priorities in areas with overlap could be reduced by entering an additional, externally prepared, now negatively weighted, interaction layer into analysis.

While pair-wise interactions between a limited number of ES can plausibly be treated case by case using the techniques described above, the situation becomes more difficult when there are many ES and interactions: getting proper parameter estimates will most likely be difficult unless credible estimates happen to be available via earlier analysis. In addition, dealing with higher-order interactions between ES will be hard due to the large numbers of such interactions, difficulties with parameterization, and complicated implementation.

## Discussion and conclusions

The Millennium Ecosystem Assessment has documented the importance of ecosystem services to human well-being (MA 2005). Assessment of ecosystems and their services is one of the key actions of the European Union’s (EU) Biodiversity Strategy to 2020 (European Commission 2011). Human well-being is increasingly often linked to ES via the concepts of ecosystem and human health and green infrastructure (Haines-Young and Potschin 2009; Liquete et al. 2015). Thus, ES become strongly linked to general land use planning (de Groot et al. 2010; Reyers et al. 2010). Multi-functionality is a concept that is constantly associated to ES and green infrastructures (Lafortezza et al. 2013). It simply means that one area can provide multiple benefits in terms of ES and possibly for biodiversity as well, thereby linking green infrastructures to SCP. Given this level of interest, there is need to understand how multiple ES should be treated in spatial prioritization together with other considerations, including distributions of biodiversity features, costs and threats.

Based on the present analysis, inclusion of ES in SCP is not as straightforward as adding ES potential layers as standard features into prioritization. The main complication is that ES may have at least three types of connectivity requirements; local area size and network-type connectivity requirements for provision, connectivity flow requirements between ES demand and supply, and large-scale requirements for equitable distribution of ES across multiple stakeholders (regions, administrations, countries, etc.). Here, we have outlined technical solutions for how such connectivity responses might be implemented using the Zonation approach and software for spatial prioritization (Fig. 1; Table 1). Some of these techniques should be applicable with other software packages as well. That said, there are considerations for which perfect solutions do not exist, at least not in the general-purpose prioritization approaches available in Zonation (Lehtomäki et al. 2016). For example, due to practical limitations imposed by large numbers of features in SCP analysis, it is only partially possible to account for interactions between features.

The present work impinges upon the debate about whether biodiversity and ES should be treated together or separately in spatial prioritization (Chan et al. 2011; Cimon-Morin et al. 2013, 2016). Our opinion about this topic is clear: do analysis both jointly and separately, and then compare solutions. If serious tradeoffs between ES and biodiversity exist and resources are limited, it becomes a matter of preference and negotiation to decide about the most appropriate balance between biodiversity and ES. Also, it is always important to remember that selection and quality of data must be considered when interpreting prioritization results.

This work has focused on the treatment of connectivity for ecosystem services in spatial conservation prioritization, which is a previously largely untreated component of spatial conservation prioritization and ecologically based land use planning. Techniques summarized here can be of utility, for example, in land use zoning and in the development of spatial plans for green infrastructures.
